# The significance of reverse flow in ductus venosus between sixteen and twenty weeks’ gestation

**DOI:** 10.4274/tjod.61482

**Published:** 2017-03-15

**Authors:** Gökhan Karakoç, And Yavuz, Serenat Eriş Yalçın, Mehmet Özgür Akkurt, Nuri Danışman

**Affiliations:** 1 Etlik Zübeyde Hanım Women’s Health Training and Research Hospital, Clinic of Perinatology, Ankara, Turkey; 2 Süleyman Demirel University Faculty of Medicine, Department of Perinatology, Isparta, Turkey; 3 Zekai Tahir Burak Women’s Health Training and Research Hospital, Clinic of Perinatology, Ankara, Turkey

**Keywords:** Ductus venosus Doppler, gestational hypertension, Gestational diabetes mellitus, second trimester, trisomy

## Abstract

**Objective:**

To evaluate the correlation between reversed a-wave in ductus venosus at 16-20 weeks’ gestation and trisomy 21 and adverse perinatal outcomes.

**Materials and Methods:**

Our study included 174 pregnant women who were under follow-up at a tertiary center between May and September 2010. Ductus venosus Doppler (DVD) measurements were obtained throughout the 6-month period from women who underwent amniocentesis procedures due to increased risk for trisomy 21 in terms of first or second trimester screening test results. These women were followed up for enrollment of subsequent data about perinatal outcomes.

**Results:**

In 13 of 174 cases, Doppler studies indicated a reversed a-wave in the ductus venosus. Of these fetuses, 3 were diagnosed as having trisomy 21 after amniocentesis, which related to 60% (3 of 5 fetuses) of all fetuses with trisomy 21. The pregnant women with reversed a-wave in DVD also had an increased rate of preeclampsia (15%) and gestational diabetes mellitus (GDM) (23%) in late pregnancy.

**Conclusion:**

Reversed a-wave in ductus venosus between 16-20 weeks’ gestation is associated with increased risk of trisomy 21, preeclampsia, and GDM. If further prospective studies confirm its utility, DVD interrogation for trisomy 21 may be extended until 20 weeks’ gestation.

## INTRODUCTION

The primary purpose of prenatal aneuploidy screening tests is early detection of pregnancies at high risk for Down syndrome, which is the most common autosomal trisomy among live births^(1)^. First trimester combined screening tests can detect approximately 84% of trisomy cases with a false positive rate (FPR) of 5 percent^(2)^. Incorporation of assessment of nasal bone, tricuspid blood flow, and ductus venosus (DV) waveform to the combined test increases the detection rate to approximately 93-96% with a FPR of 2.5 percent. In the second trimester, by combining classic ultrasonographic markers for Down syndrome (hyperechogenic intestines, an echogenic cardiac focus, pyelectasis, and short femur or humerus) with maternal age can diagnose 70% of trisomy 21 cases, but it may also record a false positive diagnosis in 10-15% of cases^(3)^.

DV is a special shunt that directs oxygen rich blood from the umbilical vein to the heart^(4)^. The characteristic properties of ductal blood flow include a high flow rate during ventricular systole (S wave) and diastole (D wave), and a continuous forward flow during atrial systole (A wave). A-wave negativity is regarded as a reflection of fetal cardiac dysfunction^(5)^.

Abnormal DV waveforms between 10 to 13^+6^ weeks have shown a relationship with chromosomal defects, cardiac anomalies, and poor gestational prognosis^(5)^. Following the demonstration of a correlation between abnormal ductal flow and nuchal translucency (NT), it has been proposed that a combined assessment of DV and NT thickness may increase the efficacy of early sonographic screening of trisomy 21. Ultrasonographic evaluations in the first trimester have shown that this abnormality is present in 66.3% to 80% of trisomy 21 cases^(6)^. Additionally, DV flow abnormality is a common finding in the presence of a cardiac defect in fetal Doppler examination in the first trimester^(7,8)^. Furthermore, some abnormalities have been found in DVD ultrasonography during pregnancies with gestational hypertension (GHT), preeclampsia, and gestational diabetes mellitus (GDM)^(9,10,11)^.

Although analysis of free fetal DNA in maternal blood flow for screening is a promising method, its widespread use is still restricted due to high costs. Thus, parameters that aid increased detection rates of routine screening tests are still substantial. The aim of our study was to determine whether DV waveform abnormality in Doppler ultrasonography would aid in second trimester screening when the detection rate is relatively low, and whether it was correlated with maternal complications.

## MATERIALS AND METHODS

Our study included 174 pregnant women aged 20 to 45 years who were followed up at Zekai Tahir Burak Women’s Health Training and Research Hospital between May 2010 and September 2010. The study design was approved by the local institutional ethics committee and review board (approval number: 04/2009-16). Written consent for participation was obtained prior to recruitment into the study. The subjects were referred for high risk for Down syndrome and scheduled for amniocentesis as a result of a risk assessment based on maternal age and/or the results of double test [NT, nasal bone hypoplasia, and beta human chorionic gonadotropin (βhCG) and pregnancy-associated plasma protein A measurements between 11 and 13^+6^ gestational weeks] or triple test (βhCG, unconjugated estriol, and alpha-fetoprotein measurements between 16 and 19 gestational weeks). All pregnancies were between 16 and 20 gestational weeks. High-risk pregnancies including multiple pregnancies, maternal diabetes, and hypertension were excluded.

DVD was performed to all subjects prior to the amniocentesis procedure. Ultrasonographic examination and measurements were conducted by two sonographers (G.K. and A.Y.) using a Voluson 730 Expert color Doppler ultrasonography device. A 4 MHz convex transducer was used in all examinations. Doppler ultrasonography was performed in the right ventral part of the fetal body on the mid-sagittal plane. Pulsed Doppler was used for measurements from the mid-section of ductus venosus. After adjustment of the insonation angle to <30 °C, DV was easily visualized using the aliasing phenomenon. Abnormal DV blood flow was defined as reversed velocities during atrial contraction (A-wave). The demographic data of the patients, and results of amniocentesis and detailed ultrasonography were recorded. The women were followed up throughout pregnancy and gestational complications were investigated. Neonatal records were inspected after birth. All study data were digitally recorded and analyzed using Statistical Packages for the Social Science (SPSS) version 11.5. All variables are expressed as mean ± standard deviation, frequency, and percentage. All comparisons were performed using the Mann-Whitney U test. Nominal and ordinal variables were analyzed using one of the chi-square tests suitable for expected values and frequencies of the variables (Fisher’s exact, and Yates’s chi-square tests). Statistical significance was set at p<0.05.

## RESULTS

One hundred seventy-four women were enrolled in the study. The mean maternal age was 32.15 years; the mean number of previous pregnancies was 3.06; the mean number of previous deliveries was 2.24; and the mean number of previous abortions was 0.75. The demographic data of patients are shown in Table 1.

Amniocentesis was scheduled according to the results of first trimester screening in 66 patients and the results of second trimester screening in 98 patients. Twenty-four (13.8%) subjects were aged more than 35 years. Of these, six women had detected risk in the first screening test and eight had risk in the second trimester screening test. The remaining 10 women were offered amniocentesis due to maternal anxiety.

In the later stages of pregnancy, 6 (3.4%) women developed GDM, 4 (2.3%) developed GHT, 4 (2.3%) developed preeclampsia, and 2 (1.1%) developed abortus. Eleven (68.8%) of 16 pregnancies with maternal complications had normal DVD and 5 (31.2%) had abnormal DVD. Three (50%) of these women had GDM and 2 (50%) had preeclampsia. The presence of reversed a-wave in DV was a significant predictor of maternal complications in later weeks of pregnancy (p=0.003, Fisher’s exact test) (Table 2).

Among the pregnant women without fetal problems (spontaneous abortus, trisomy, cardiac defect), 157 (95.7%) had normal DVD and 7 (4.3%) had reversed a-wave in DV. A total of 10 fetuses had problems, 2 of which were lost to spontaneous abortion (post-amniocentesis abortus), 4 had cardiac anomalies, and 5 had trisomy 21 (1 fetus had both trisomy 21 and ventricular septal defect).

In 13 of 174 cases, Doppler studies indicated a reversed a-wave in the ductus venosus. Of those, 3 fetuses had trisomy 21, which related to 60% (3 of 5 fetuses) of all fetuses with trisomy 21. In the euploid group, reversed flow in the DV was observed in 5.9% of the cases. The rate of reversed a-wave in DV was significantly higher than in normal DVD in fetuses with trisomy 21 (p=0.003, Fisher’s exact test) (Table 3).

## DISCUSSION

The diagnostic accuracy and false positivity rates of the available screening methods for trisomy 21 have not reached an ideal level^(12,13)^. Recently, a novel screening method called non-invasive prenatal test based on free fetal DNA analysis in maternal blood was developed^(14,15)^. However, such tests cannot be included in routine screening tests owing to their high costs in developing countries.

Based on fetal ultrasonographic examinations of cases of trisomy 21, former studies reported that DV showed an abnormal flow pattern in these cases^(16,17,18)^. In a study on over 5000 pregnant women, 281 cases of trisomy 21 were diagnosed. An abnormal flow pattern in DVD was observed in 80% of trisomy 21 cases and 5% of euploid fetuses^(12)^. Another study detected a DV flow pattern abnormality in the first trimester in 66.3% of trisomy 21 cases^(6)^. In most studies where DV was assessed with Doppler ultrasonography, the assessment was made in the first trimester. Geipel et al.^(16)^ evaluated women with 808 euploid fetuses and 37 fetuses with Down syndrome between the 14^th^ and 18^th^ weeks of gestation. These women were investigated for the presence of abnormal DV waveform, tricuspid regurgitation, and nasal bone hypoplasia/aplasia. The trisomy 21 group had reversed a-wave in DVD at a rate of 23.3%, tricuspid regurgitation 27%, and nasal bone hypoplasia/aplasia 45.9%, and the euploid group had a rate of 1.6% for reversed a-wave in DVD, 4.6% for tricuspid regurgitation, and 3.2% for nasal bone hypoplasia/aplasia. The authors concluded that the presence of these ultrasonographic markers in the second trimester increased the risk of Down syndrome by 6-15-fold. Combining maternal age, nuchal fold, nasal bone, tricuspid regurgitation with DVD, they accurately diagnosed 90% of all trisomy 21 cases^(16)^. A large-scale study conducted in 2011 that involved 20,000 euploid fetuses and 20,000 cases of trisomy 21 showed that tests including DV, tricuspid blood flow, and nasal bone at 15-18 gestational weeks improved trisomy 21 screening performance when used in conjunction with maternal age. That study revealed rates of 1.7% and 14.3% for abnormal DV flow in the euploid and trisomy 21 groups, respectively^(17)^.

We detected DV waveform abnormalities in 3 (60%) of 5 cases of trisomy 21. Our study indicated that DVD evaluation at 16-20 gestational weeks was significantly beneficial for predicting trisomy 21, in accordance with the above-mentioned studies. The strengths of our study over other studies include its prospective design and the more homogenous nature of its sample.

### Study Limitations

Our limitation is the small sample size. It has been shown previously that among cases proven to be free of chromosomal anomalies in first trimester fetal Doppler ultrasonography examination, abnormal DV flow pattern was present in 0-40% of those without cardiac defects, but 75-100% of those with cardiac defects^(7,8)^. We found that 3 of 4 fetuses with cardiac problems had abnormal DV waveform. This rate appears to be in accordance with previously reported studies.

It has been reported that women with GHT and preeclampsia had altered DV waveforms in fetal Doppler ultrasonographic examination performed after the index of disorder due to placental dysfunction^(9)^. A recent study that aimed to determine the performance of maternal characteristics, Doppler, and a set of biochemical markers for preeclampsia screening in the first and second trimester demonstrated that inclusion of pulsatility index of DV to other parameters could potentially aid patient counseling with regard to early screening for preeclampsia^(10)^. We demonstrated abnormal DV flow in all 4 cases of preeclampsia. Some studies indicated that the pulsatility index of DV was higher in women with GDM compared with control subjects^(11,18)^. We found an abnormal DV flow pattern in 3 of 6 women with gestational diabetes. Our results concerning GDM were in agreement with literature reports. In our study, the ratio of DVD abnormality was significantly greater in pregnancies that would later develop maternal complications.

## CONCLUSION

Despite limited safety data due to the small number of fetuses with Down syndrome, we are of the opinion that the addition of DVD to second trimester screening tests will be an inexpensive and beneficial method to increase the rate of Down syndrome detection in women who cannot undergo DVD examinations in the first trimester. DVD examination in the second trimester may also provide information to predict adverse pregnancy outcomes such as preeclampsia and GDM. However, this hypothesis needs to be supported by further research with large sample sizes.

## Figures and Tables

**Table 1:**
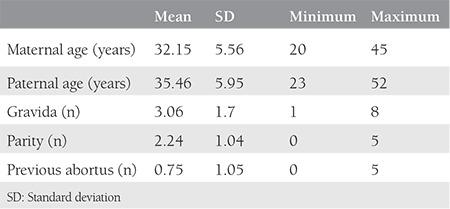
The demographic data of the patients

**Table 2:**
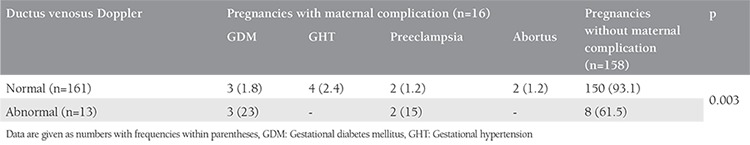
Correlation between maternal complications and ductus venosus Doppler

**Table 3:**
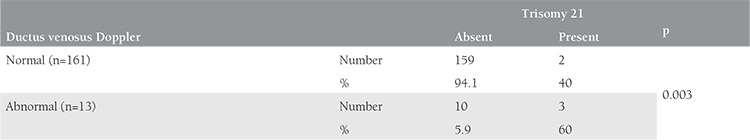
Distribution of normal and abnormal ductus venosus flow rate waveforms based on the presence of Down syndrome
